# Low-dose menaquinone-4 improves γ-carboxylation of osteocalcin in young males: a non-placebo-controlled dose–response study

**DOI:** 10.1186/1475-2891-13-85

**Published:** 2014-08-27

**Authors:** Eriko Nakamura, Mami Aoki, Fumiko Watanabe, Ayako Kamimura

**Affiliations:** Healthcare Products Development Center, KYOWA HAKKO BIO CO., LTD, 2 Miyukigaoka, Tsukuba, Ibaraki 305-0841 Japan

**Keywords:** Menaquinone-4, Osteocalcin, Vitamin K

## Abstract

**Background:**

Menaquinone-4 is a type of vitamin K that has a physiological function in maintaining bone quality via γ-carboxylation of osteocalcin. However, little is known about the beneficial effect of intake of dosages below1500 μg/day.

**Findings:**

Fifteen healthy males aged 25.0 years (median) participated in a non-placebo-controlled dose-examination study. They received menaquinone-4 daily for 5 weeks at 0, 300, 600, 900, and 1500 μg/day in weeks 1, 2, 3, 4, and 5, respectively. Compared with baseline, serum γ-carboxylated osteocalcin levels were significantly greater at an intake of 900 μg/day or more; serum undercarboxylated osteocalcin levels and the ratio of serum undercarboxylated osteocalcin to γ-carboxylated osteocalcin were significantly lower than baseline at doses of 600 μg/day or more.

**Conclusions:**

This preliminary graded-dose study suggested that menaquinone-4 supplementation at 600 μg/day or more is likely to be important in terms of vitamin K requirements for bone health.

## Finding

### Introduction

There are two major forms of dietary vitamin K: phylloquinone and menaquinone. Vitamin K plays an important role in coagulation and bone homeostasis as a coenzyme that mediates γ-carboxylation of glutamate residues into γ-carboxylated proteins such as coagulation factors, osteocalcin, and matrix Gla-protein. The precise role of osteocalcin is not known, but it is known that γ-carboxylated osteocalcin (Gla-OC) is a bone protein that attracts Ca^2+^ and incorporates it into hydroxyapatite crystals. In contrast, undercarboxylated osteocalcin (ucOC) cannot bind to hydroxyapatite [1]. Serum Gla-OC is used as a marker of bone formation, whereas serum ucOC is used as a marker of vitamin K deficiency and as a predictor of hip fracture risk independently of femoral neck bone mineral density [2]; the cut-off value of 4.5 ng/mL for ucOC has been validated [3] and is generally accepted in the selection of Japanese patients for medication for osteoporosis. However, absolute Gla-OC or ucOC levels are not indicative of the overall vitamin K status in the bone metabolism. The ratio of ucOC to Gla-OC (UGR) is therefore used as a sensitive marker of the vitamin K status of bone [4]. Overall, vitamin K supplementation controls bone homeostasis by increasing Gla-OC levels and decreasing ucOC levels and the UGR.

In most countries, dietary reference intakes (DRIs) of vitamin K are based on saturation of the coagulation system (75 to 120 μg for adult males) [5, 6]. However, some epidemiological studies have suggested that vitamin K requirements for maintaining skeletal health might be higher than the current DRIs at various ages [7–10]. Furthermore, approximately 500 μg/day of dietary vitamin K by phylloquinone supplementation was needed to significantly decrease ucOC levels in substudy A [11]. An intervention study of menaquinone-4 (MK-4), the molecular weight and bone metabolic effects of which are similar to those of phylloquinone, revealed that MK-4 supplementation at 1500 μg/day for a year improved bone quality in elderly females [12]. However, this dosage of MK-4 is too high to achieve from intake in natural foods. Moreover, to our knowledge there has been only one study of the effectiveness of dosages of MK-4 below 1500 μg/day in people aged in their 30s to 50s [13].

Our aim was to clarify the effects of low dosages of supplemental MK-4 (below 1500 μg/day) on bone health in healthy young adults, as measured by serum Gla-OC and ucOC levels and the UGR.

## Methods

### Subjects

Fifteen healthy Japanese males aged 25.0 years (median) and not currently taking *natto* (fermented soybean, which has a high concentration of vitamin K) or medications were screened a week before Day 1. Subjects were not allowed to take *natto* during the study period. Subject recruitment, informed consent procedures, and all experimental methods were approved by the Oriental Ueno Health Center Ethical Committee in Tokyo and were conducted according to the principles of the Declaration of Helsinki.

### Study design

We followed the study design of Binkley *et al*. [11]. Capsules containing 300 μg MK-4 (KYOWA HAKKO BIO CO., LTD., Tokyo) were prepared and stored in light-shielded containers. Over the 5-week non-placebo-controlled study, subjects received no capsules on Days 1 to 7. This was followed by intake of 300, 600, 900, and 1500 μg/day on Days 8 to 14, Days 15 to 21, Days 22 to 28, and Days 29 to 35, respectively. The daily dose was taken during or after the evening meal, and all blood samples were taken under fasting conditions. Sera for measurement of Gla-OC (MK-128, Takara Bio Inc., Otsu) and ucOC (Picolumi ucOC, Eidia Co. Ltd., Tokyo) were drawn on Days 1, 8, 15, 22, 29, and 36 in the morning. Anthropometric parameters and an indicator of coagulation, prothrombin time – international normalized ratio (PT-INR), were measured on the screening day and on Day 36. The plasma vitamin K fraction (phylloquinone, MK-4, menaquinone-7) was assessed on Days 1, 15, and 36 by using high performance liquid chromatography. Subjects kept daily dietary vitamin K records of the amounts of vitamin K–rich food (*natto*, broccoli, bok-choy, garland chrysanthemum, spinach, Japanese mustard spinach, Egyptian spinach, Chinese cabbage, chicken) they consumed. At the blood-sampling visits every week, the daily records were collected and trained dieticians interviewed the subjects to estimate their consumption of vitamin K–rich food. Dietary vitamin K intake was calculated by using software (Excel Eiyo-kun ver. 6.0, Kenpakusha, Tokyo) and was based on the Standard Tables of Food Composition in Japan – 2010 [14]. Data were excluded from the analysis if a subject took the capsules for less than 85.7% of the 28-day supplementation period.

### Statistical analysis

Data were presented as medians with ranges (minimum, maximum). The Wilcoxon signed-rank test was used to test the anthropometric and coagulation values for statistical significance between baseline (screening day) and Day 36. The raw values of plasma vitamin K fraction and the raw values and changes in serum ucOC, Gla-OC, and UGR were compared with baseline (Day 1) by using Friedman’s test and a post hoc Bonferroni-adjusted Wilcoxon signed-rank test. A *P-*value of less than 0.05 was considered to indicate significance. Statistical analysis was conducted with JMP 11.0.0 (SAS Institute Inc., Cary, North Carolina).

## Results

Two subjects were excluded: one subject failed to meet the intake ratio criterion and the other was unable to visit for testing on Day 36. Another subject took a double dose on Day 26. Therefore, we analyzed the data from 13 subjects.

Values for anthropometric data, blood coagulation markers, and plasma vitamin K fraction are given in Table 1. Plasma phylloquinone level was significantly lower on Day 36 than at baseline, and plasma MK-4 level was significantly greater on Day 36 after graded MK-4 supplementation than at baseline. On Day 15 the plasma menaquinone-7 level was significantly lower than at baseline (data not shown). PT-INR significantly increased, but the value was within the normal range. No adverse events related to MK-4 were reported throughout the experiment period.

**Table 1:** **Anthropometric, coagulation marker, and plasma vitamin K fraction values at baseline and on Day 36**

		Units	Baseline		Day 36	
			Median	Range	Median	Range
**Anthropometric data**	Age	years	25.00	(20.00-29.00)	-	-
	Weight	kg	58.55	(50.55-68.25)	57.95	(50.50-66.90)
	BMI	kg/m^2^	20.60	(18.50-24.10)	20.10	(18.30-23.70)
**Coagulation marker**	PT-INR	-	1.05	(0.96-1.09)	1.08	(1.03-1.22)*
**Plasma vitamin K fraction**	Phylloquinone	ng/mL	0.35	(0.22-0.85)	0.20	(0.08-0.49)*
	Menaquinone-4	ng/mL	0.14	(0.10-0.17)	0.58	(0.33-1.78)**
	Menaquinone-7	ng/mL	0.43	(0.10-7.92)	0.25	(0.13-0.93)

Data are shown as medians with ranges (minimum, maximum) (n=13). Wilcoxon signed-rank test was used to study the effect of MK-4 supplementation on weight, BMI and PT-INR (comparison between baseline and Day 36 values). Plasma vitamin K fraction was analyzed by using Friedman’s test and the post hoc Bonferroni-adjusted Wilcoxon signed-rank test (comparison among baseline and Day 36 values). *P < 0.05, **P < 0.01.

Serum ucOC, Gla-OC, the UGR, and the dietary vitamin K intake after each dosage period are shown in Table 2. Serum ucOC level exceeded the cut-off value (4.5 ng/mL) in 69.23% of subjects at baseline; on Days 22, 29, and 36 it was significantly lower than at baseline. Serum Gla-OC level was significantly greater on Days 29 and 36 than at baseline. In some subjects the dietary vitamin K intake was very much lower than the DRIs. The UGR decreased dose-dependently and on Days 22, 29, and 36 was significantly lower than at baseline; vitamin K status for bone metabolism thus improved.

**Table 2:** **Serum ucOC, Gla-OC, UGR, and dietary vitamin K intake values before and after MK-4 supplementation**

Measurement day	Dosage	ucOC (ng/mL)		Gla-OC (ng/mL)		UGR		Dietary vitamin K intake (μg/week)	
		Median	Range	Median	Range	Median	Range	Median	Range
Day 1	Baseline	5.03	(2.20-23.03)	18.41	(10.62-26.60)	0.32	(0.13-1.70)	-	-
Day 8	0 μg/day	5.81	(2.63-26.65)	18.71	(8.62-24.36)	0.36	(0.12-1.88)	40.18	(0.00-540.27)
Day 15	300 μg/day	6.77	(1.89-14.52)	21.27	(10.57-26.91)	0.35	(0.18-0.54)	17.40	(0.00-638.76)
Day 22	600 μg/day	4.82	(1.84-8.73)*	25.33	(12.29-33.04)	0.22	(0.14-0.31)**	31.68	(0.00-389.40)
Day 29	900 μg/day	2.98	(1.27-6.90)**	22.75	(12.38-38.10)*	0.15	(0.10-0.23)**	9.60	(0.00-196.94)
Day 36	1500 μg/day	3.92	(1.88-7.52)*	23.33	(13.97-45.49)**	0.15	(0.13-0.21)**	7.50	(0.00-167.58)

Data are shown as medians with ranges (minimum, maximum) (n = 13). Compared with baseline, serum ucOC levels and the UGR decreased significantly, whereas serum Gla-OC levels increased significantly. *P < 0.05, **P < 0.01 (Friedman’s test and the post hoc Bonferroni-adjusted Wilcoxon signed-rank test).

The changes in serum Gla-OC levels relative to baseline increased significantly after MK-4 supplementation on Days 29 and 36 (Table 3). The changes in serum ucOC levels and the UGR decreased significantly on Days 22, 29, and 36.

**Table 3:** **Changes in serum ucOC, Gla-OC, and UGR from baseline after MK-4 supplementation**

Measurement day	Dosage	ucOC (ng/mL)		Gla-OC (ng/mL)		UGR	
		Median	Range	Median	Range	Median	Range
Day 8	0 μg/day	0.29	(-0.86-3.62)	0.00	(-4.13-6.96)	0.02	(-0.06-0.18)
Day 15	300 μg/day	0.60	(-8.51-2.05)	2.28	(-4.88-13.35)	-0.02	(-1.16-0.10)
Day 22	600 μg/day	-0.72	(-14.30-0.15)*	3.19	(-1.71-19.48)	-0.12	(-1.43-0.01)**
Day 29	900 μg/day	-2.09	(-16.13-0.05)**	1.76	(-1.04-24.54)*	-0.17	(-1.52-0.001)**
Day 36	1500 μg/day	-1.59	(-15.51-0.65)*	4.99	(0.81-31.93)**	-0.16	(-1.53-0.02)**

Data are shown as medians with ranges (minimum, maximum) (n = 13). The changes in serum ucOC levels and the UGR decreased significantly from baseline, whereas the changes in Gla-OC levels increased significantly. *P < 0.05, **P < 0.01 (Friedman’s test and the post hoc Bonferroni-adjusted Wilcoxon signed-rank test).

The effects observed here were likely derived mainly from the capsules, because the serum ucOC and Gla-OC levels were unrelated to vitamin K intake from the diet during this study, with the exception of the baseline value (data not shown).

## Discussion

We showed here in our graded-dose examination that serum Gla-OC levels increased significantly with intakes of 900 μg/day or more and serum ucOC levels and the UGR decreased significantly with intakes of 600 μg/day or more. Therefore, MK-4 supplementation at 600 μg/day after a week’s initial intake at 300 μg/day improved vitamin K status in terms of bone health, especially on the basis of serum ucOC level as a marker of vitamin K deficiency and the UGR as a sensitive marker of γ-carboxylation of osteocalcin.

Dietary vitamin K intake tends to be low in the British population [15] and to be less than the adequate intake in the DRIs in North America [5]. Currently, the Japanese DRIs is 60 μg/day in females, and 75 μg/day in males, aged 18 to 29 years [6]. However, in 2015 the Japanese DRIs will be revised upward to 150 μg/day for both males and females aged 18 to 29 years [16]. The average dietary vitamin K intake in Japanese people in their 20s (201 μg/day) [17] satisfies the current and proposed future DRIs. In contrast, the dietary vitamin K intakes of some of our subjects were very much lower than the DRIs and the Japanese average dietary vitamin K intake. Notably, also, plasma phylloquinone and menaquinone-7 levels decreased during the study period, possibly reflecting decreases in dietary vitamin K intake on Days 15, 29, and 36, because dietary vitamin K consists mainly of phylloquinone (leafy green vegetables) and menaquinone-7 (fermented foods). The serum ucOC levels at baseline (Table 2) indicated that our subjects tended to have vitamin K deficiency, and an additional MK-4 intake of 600 μg/day or more was needed to reduce serum ucOC levels to close to the cut-off value (4.5 ng/mL) [3]. Depending on dietary habits, therefore, intakes of vitamin K–rich food, or vitamin K supplementation, exceeding the DRIs may have been needed to improve bone health in our intervention study. Vitamin K status at baseline might affect γ-carboxylation of osteocalcin in response to supplementary vitamin K [18]; this could be one reason why our effective dose was lower than that in the report by Takeuchi *et al.* [13]. Those authors found that supplementation with MK-4 at 1500 μg/day for 4 weeks significantly improved vitamin K status in healthy males, but supplementation with MK-4 at 500 or 1000 μg/day did not produce a clear difference compared with placebo.

Our study had some limitations. Because it was a graded-dose study, each preceding, lower, dose may have affected the subsequent serum ucOC and Gla-OC levels and the UGR. Furthermore, because the assessment items for dietary vitamin K intake were limited to nine vitamin K–rich foods, the minimum dietary vitamin K intake was 0 and the dietary vitamin K intake tended to decrease during the study period. To make the dietary assessment more closely reflect the entire dietary vitamin K intake, in future we will have to select more sophisticated methods: for example, we may need to estimate each participant’s dietary intake of not only vitamin K but also other nutritional components [9, 10]. We will also need to investigate the lowest effective dose by measuring other bone metabolism parameters in a randomized, double-blind, placebo-controlled study that considers the dietary habits and genotypes of the subjects [19].

In conclusion, in this graded-dose study, we showed that supplementation with MK-4 at 600 μg/day or more is likely to be important in terms of vitamin K requirements for bone health. These relatively low MK-4 dosages (below 1500 μg/day) may improve bone formation and reduce bone fracture risk.

### Available supporting data

Supplementary online material has not been submitted.

## Abbreviations

DRIs: Dietary reference intakes
Gla-OC: γ-carboxylated osteocalcin
MK-4: Menaquinone-4
PT-INR: Prothrombin time‒international normalized ratio
UGR: Ratio of ucOC to Gla-OC
ucOC: Undercarboxylated osteocalcin.
